# Use of specific antimicrobials for COVID-19: should we prescribe them now or wait for more evidence?

**DOI:** 10.1136/postgradmedj-2020-137990

**Published:** 2020-05-21

**Authors:** Abdullah AlAkhras, Ahmed Husein AlMessabi, Hala Abuzeid, Saye Khoo, Emmanuel Fru Nsutebu

**Affiliations:** Internal Medicine Department, Sheikh Shakhbout Medical City, Abu Dhabi, United Arab Emirates; Infectious Disease Division, Sheikh Shakhbout Medical City, Abu Dhabi, United Arab Emirates; Critical Care Department, Sheikh Shakhbout Medical City, Abu Dhabi, United Arab Emirates; Faculty of Health and Life Sciences, University of Liverpool, Liverpool, UK; Infectious Disease Division, Sheikh Shakhbout Medical City, Abu Dhabi, United Arab Emirates

**Keywords:** infectious diseases, therapeutics

As the number of COVID-19 cases worldwide rises exponentially, clinicians and healthcare systems are faced with a difficult dilemma. Should they focus on supportive care and wait for the results of clinical trials or join other clinicians who are already prescribing specific antimicrobials which may be active against the virus?

Eighty per cent of patients with COVID-19 have mild infection, 15% develop severe illness and 5% require critical care admissions for organ support.^1^ Mortality is currently estimated to be 1%–3%.^1^ Older patients (>80 years old) and patients with comorbidity such as respiratory disease, diabetes, stroke, cardiac disease and cancer are more likely to die from the illness.^2^ Mortality for a patient admitted to critical care is estimated to be about 50%; however, this may be dependent on access to and quality of critical care services. So far, no vaccine or drug has been approved to treat human coronaviruses including COVID-19. In addition, there is no validated clinical tool or test to predict patients who are likely to develop complications.

There are currently multiple ongoing trials of specific antimicrobials for treatment and prevention of COVID-19. The most common agents proposed are hydroxychloroquine, chloroquine, ritonavir boosted lopinavir, favipravir, remdesivir, beta interferon and tocilizumab.^3^ These agents have been proposed as specific or adjuvant therapy for COVID-19 based on previous in vitro evidence of activity against severe acute respiratory syndrome coronavirus (SARS-CoV) 1 and recent evidence of in vitro activity against SARS-CoV2.^4  5^ In support of their use, there have been case reports and observational studies of patients treated with these therapies who have improved and survived.^6^ In addition, a small-sized controlled trial from France suggested significant improvements in viral load in patients treated with hydroxychloroquine and azithromycin compared with supportive care.^7^ We eagerly wait to see whether other large trials will validate the results from this study. Interestingly, despite suggestions of good in vitro activity a recently published trial of use of ritonavir boosted lopinavir in patients from China did not show in improvement in outcomes.^8^ This highlights the importance of doing and waiting for results of well conducted clinical trials before widespread use of these agents.

Anecdotal and uncontrolled data have suggested that early treatment with specific antimicrobials may reduce the risk of patients developing severe complications. This has prompted empirical use of these drugs outside of clinical trials in the hope of preventing patients from developing critical illness—for example, use of hydroxychloroquine or chloroquine as pre-exposure prophylaxis for at-risk contacts or healthcare workers. The desire to ‘do something’ for patients and colleagues is entirely understandable in the current pandemic, where conventional treatments have seemed so powerless. Moreover, some argue that drugs such as chloroquine have been used to treat infections such as malaria and HIV for many years with minimal safety concerns.^4^ Some of these antimicrobials have also been included in national treatment guidelines or approved for use by some regulatory authorities. For example, US and French authorities have already authorised the use of hydroxychloroquine despite the limited evidence. All these reasons may encourage clinicians to prescribe these antimicrobials without restrictions.

We know that supportive therapy is currently recommended as the mainstay of treatment for COVID-19. Epidemiological data suggest low mortality rates when high-quality supportive care is provided for patients with COVID-19.^2^ Justifying use of experimental treatments outside of clinical trials for the majority of patients who have mild disease or no symptoms seems to us overly aggressive, especially if they are given at high doses which may increase the risk of serious side effects such as hepatotoxicity and cardiotoxicity. Patients with multiple morbidities are also at increased risk of drug interactions.^9  10^

We believe that the widespread use of unproven drugs in desperation to vulnerable individuals may cause harm and discourage patients and healthcare providers from participating in clinical trials. Without a control group to compare, it is impossible to know whether use of these drugs helps or causes harm. We, therefore, strongly recommended that novel or repurposed use of these is only done on compassionate grounds or as part of an ethically approved clinical trial such as the WHO-Solidarity trial. Despite the pressure to do all possible to save lives during the COVID-19 pandemic, we recommend that clinicians and especially infectious disease physicians and clinical pharmacists should continue to promote and champion antimicrobial stewardship. We would also like to stress the importance of participating in well-designed trials as the only way to save lots of lives.

## References

[1] Wu Z, McGoogan JM. Characteristics of and Important Lessons From the Coronavirus Disease 2019 (COVID-19) Outbreak in China: Summary of a Report of 72 314 Cases From the Chinese Center for Disease Control and Prevention. JAMA 2020;323:1239–42. 10.1001/jama.2020.2648 32091533

[2] Huang C, Wang Y, Li X, et al. Clinical features of patients infected with 2019 novel coronavirus in Wuhan, China. Lancet 2020;395:497–506. 31986264 10.1016/S0140-6736(20)30183-5 PMC7159299

[3] WHO . Landscape analysis of candidate therapeutics for COVID-19. Available: https://app.powerbi.com/view?r=eyJrIjoiNjQxZWZhOTItYzU1ZS00Y2QxLWE1ODAtOTViZjhmNjEyZjNiIiwidCI6ImY2MTBjMGI3LWJkMjQtNGIzOS04MTBiLTNkYzI4MGFmYjU5MCIsImMiOjh9.htm [Accessed 10 Apr 2020].

[4] BMJ . Best practice COVID-19, BMJ best practice, 02 March 2020. Available: https://bestpractice.bmj.com/topics/en-gb/3000168/guidelines.htm [Accessed 10 Mar 2020].

[5] Wang M, Cao R, Zhang L, et al. Remdesivir and chloroquine effectively inhibit the recently emerged novel coronavirus (2019-nCoV) in vitro. Cell Res 2020;30:269–71. 10.1038/s41422-020-0282-0 32020029 PMC7054408

[6] Cortegiani A, Ingoglia G, Ippolito M, et al. A systematic review on the efficacy and safety of chloroquine for the treatment of COVID-19. J Crit Care 2020. doi:10.1016/j.jcrc.2020.03.005. [Epub ahead of print: 10 Mar 2020]. PMC7270792 32173110

[7] Gautret P, Lagier J-C, Parola P, et al. Hydroxychloroquine and azithromycin as a treatment of COVID-19: results of an open-label non-randomized clinical trial. Int J Antimicrob Agents 2020:105949. 10.1016/j.ijantimicag.2020.105949 32205204 PMC7102549

[8] Cao B, Wang Y, Wen D, et al. A trial of lopinavir-ritonavir in adults hospitalized with severe COVID-19. N Engl J Med 2020;382:1787–99. 10.1056/NEJMoa2001282 32187464 PMC7121492

[9] Guo Y-R, Cao Q-D, Hong Z-S, et al. The origin, transmission and clinical therapies on coronavirus disease 2019 (COVID-19) outbreak – an update on the status. Mil Med Res 2020;7:1–10. 10.1186/s40779-020-00240-0 32169119 PMC7068984

[10] Lai C-C, Shih T-P, Ko W-C, et al. Severe acute respiratory syndrome coronavirus 2 (SARS-CoV-2) and coronavirus disease-2019 (COVID-19): the epidemic and the challenges. Int J Antimicrob Agents 2020;55:105924. 10.1016/j.ijantimicag.2020.105924 32081636 PMC7127800

